# Phytochemical Characterization of Germinated Mung Bean Sprout (*Vigna radiata* L., cv. Sanpo) and Anti-Inflammatory Activity In Vitro and Preliminary Effects in a Rat Periodontitis Model

**DOI:** 10.3390/foods15213910

**Published:** 2026-10-31

**Authors:** Ji Yeong Yang, Sang Hoon Lee, Yeong Jae An, Se Won Cho, Seung Yeob Song

**Affiliations:** 1 Department of Clinical Laboratory Science, Dongnam Health University, Suwon 16328, Republic of Korea; 2 Food Tech Resources Research Division, Department of Food Sciences, National Institute of Crop and Food Science, Wanju 55365, Republic of Korea

**Keywords:** mung bean sprout, *Vigna radiata*, sanpo cultivar, extraction optimization, periodontitis, anti-inflammatory activity

## Abstract

Periodontitis is a chronic inflammatory oral disease characterized by mucosal barrier destruction and alveolar bone loss. This study characterized germinated mung bean sprouts (*Vigna radiata* L.) and evaluated the biological activity of the resulting extract in vitro and in vivo. Among six domestic cultivars, ‘Sanpo’ showed the highest total phenolic content (comparable to ‘Jinhwang’) and the highest ABTS radical scavenging activity (74.85%). Across Days 7–17 of germination, Day 10 sprouts contained the highest rutin level while quercetin was still detectable; quercetin was no longer detected at Day 17. In an analytical screen, extraction with 30% ethanol at 50 °C gave the highest recovery of rutin, quercetin, and kaempferol. A room-temperature 30% ethanol extract (MSE) was non-cytotoxic (>90% viability at 100 μg/mL) and significantly reduced IL-6 and IL-8 release in RAW264.7, HaCaT, and YD-38 cells. In a *Porphyromonas gingivalis* LPS-induced periodontitis rat model (n = 7 per group), LPS significantly increased the periodontal inflammation score, whereas MSE (50 mg/kg/day) produced only non-significant numerical reductions in alveolar bone loss and inflammation relative to the LPS group. These in vivo findings are preliminary and hypothesis-generating. Sanpo sprout extract warrants further investigation as a candidate ingredient for oral care and functional food applications.

## 1. Introduction

Natural bioactive secondary metabolites isolated from crops and their seedlings have attracted sustained research interest owing to their wide range of biological properties, including antioxidant, anti-inflammatory, and antimicrobial effects [1,2,3]. Among the many food crops examined for such bioactive potential, mung bean (*Vigna radiata* L.) and its germinated sprouts stand out as an inexpensive, widely consumed raw material rich in polyphenols and flavonoids, with documented antioxidant, anti-inflammatory, and metabolic health benefits [4,5]. Despite this recognized value, systematic studies that integrate genotype selection, harvest-time optimization, and extraction-condition screening into a single pipeline—and then carry that material through to a specific, disease-relevant functional validation—remain comparatively rare for mung bean sprouts, in much the same way that such integrated approaches have only recently been applied to other seedling crops such as oat and wheat [6,7].

Periodontitis affects a substantial proportion of the adult population worldwide [8]. A growing body of evidence indicates that the flavonoid triad most closely associated with mung bean sprouts—rutin, quercetin, and kaempferol—possesses biological activities directly relevant to bacterial-induced inflammatory disease [9,10,11]. Flavonoids of this class have been shown to suppress gingipain activity and biofilm formation by *Porphyromonas gingivalis*, the principal etiological agent of periodontitis [1], and to attenuate lipopolysaccharide (LPS)-induced nuclear factor-kappa B (NF-κB) and mitogen-activated protein kinase (MAPK) signaling in macrophage models [12,13]. In a related but mechanistically instructive example, kaempferol has been shown to reduce pro-inflammatory cytokine expression (TNF-α, IL-1β, IL-8) and to suppress the translocation of the virulence factors CagA and VacA in gastric epithelial cells infected with *Helicobacter pylori* [14]—a different Gram-negative pathogen, but one that illustrates the same general principle of a dietary flavonoid dampening both bacterial virulence-factor delivery and the downstream host inflammatory cascade. Collectively, these findings support the biological plausibility of a flavonoid-triad-rich mung bean sprout extract as a candidate agent against *P. gingivalis*-driven periodontal inflammation, motivating the present work. Accordingly, rutin, quercetin, and kaempferol were chosen as marker compounds for harvest-time profiling, whereas total phenolic and flavonoid contents (TPC and TFC) were used only as a screening tool for cultivar selection and were not tracked during germination.

Realizing this potential, however, requires addressing three sources of phytochemical variability that are frequently left uncontrolled in the mung bean literature. First, germination itself is well established as a bioprocessing step that substantially increases total phenolic and flavonoid content relative to the ungerminated seed [15,16], but the accumulation of individual target flavonoids during germination is not monotonic with growth time. Studies profiling glycosylated flavonoids across a full harvest-time series in other seedling crops—for example, oat seedlings monitored across six harvest times (5–15 days) [6] and wheat seedlings monitored across five harvest times (5–14 days) [7]—have consistently shown that total and individual metabolite contents peak at an intermediate harvest time rather than increasing steadily with plant maturity, and that this pattern is compound-specific rather than uniform across the whole phytochemical profile. This is consistent with the specific case of the rutin–quercetin–kaempferol triad, in which quercetin is generated transiently via glycosidase-mediated cleavage of rutin’s glycosidic bond and is subsequently depleted [17], making the precise harvest window a critical, compound-specific determinant of extract quality rather than a matter of “the later the better.”

Second, genotype contributes an independent layer of variability: domestically bred cultivars of a given crop can differ substantially in baseline phenolic/flavonoid density and in bioactive-compound content even under identical growth conditions, as shown for oat seedling cultivars screened in parallel at each harvest time [6], and cultivar rankings can further depend on which antioxidant assay is used, since DPPH and ABTS radical-scavenging assays probe distinct chemical mechanisms (single electron transfer versus mixed hydrogen-atom/electron transfer, respectively) and do not always rank samples identically [18]. Third, extraction solvent polarity and processing temperature jointly determine the recovery efficiency of the rutin–quercetin–kaempferol triad, with the more polar glycoside and less polar aglycone fractions responding differently to hydroethanolic solvent systems and thermal input [19].

Despite the individual recognition of these harvest-time, cultivar, and extraction-condition effects in related seedling crops, an integrated pipeline that carries a mung bean cultivar through all three steps and then functionally validates the resulting extract—first across multiple physiologically relevant in vitro cell barriers, and subsequently in a *P. gingivalis* LPS-induced periodontitis animal model—has not, to our knowledge, been reported. To address this gap, the present study (i) comparatively screens six domestic mung bean cultivars to select the genotype with the most favorable combined phenolic/flavonoid and antioxidant profile; (ii) profiles the rutin–quercetin–kaempferol triad across a 17-day germination window to identify a harvest time at which all three compounds are present at high levels; (iii) systematically screens extraction solvent and temperature conditions to identify extraction conditions giving high recovery of the target flavonoids; and (iv) evaluates the room-temperature extract (MSE) for cytocompatibility and anti-inflammatory activity across immune, epidermal, and gingival cell models, followed by a pilot in vivo evaluation in a *P. gingivalis* LPS-induced periodontitis rat model. Together, these steps aim to provide a characterized workflow from cultivar and harvest-time selection to extraction as a basis for evaluating Sanpo mung bean sprout extract as a candidate ingredient for oral-care and functional food applications.

## 2. Materials and Methods

### 2.1. Plant Materials and Cultivation

The secondary metabolite profile of mung bean sprouts is sensitive to environmental variables and developmental stage. Cultivation conditions and harvest time-points were therefore kept constant across batches.

Six domestic cultivars of mung bean (*Vigna radiata* L.)—Dahyeon, Sanpo, Jinhwang, Samhwang, Seonhwa, and Jangan—were obtained from the National Institute of Crop Science (RDA, Wanju, Republic of Korea) and subjected to comparative screening. For each cultivar, three independent batches of seeds were germinated in a controlled biological growth chamber maintained at a constant 25 °C. Seedlings were exposed to biological white light (LED source) at a constant intensity of 40,000 lux. To identify the peak biosynthetic accumulation of the target flavonoid triad—rutin, quercetin, and kaempferol—harvesting was performed at four specific intervals: Days 7, 10, 14, and 17, with three biological replicates per timepoint. Based on preliminary screening data, the 10-day harvest was selected because it gave the highest rutin level while quercetin remained detectable (Section 3.2).

Post-harvest, the sprouts were immediately stabilized via freeze-drying using a freeze dryer (MLU-9009, Mareuda, Gwangju, Republic of Korea). The dried biomass was mechanically reduced to a fine, homogeneous powder using a high-speed grinder (WC-3L, Wonder Crusher, Osaka, Japan) and stored cryogenically at −20 °C until further analysis. These materials were used for all subsequent extraction and biological experiments.

### 2.2. Solvent Extraction Screening and Extract Preparation

To identify extraction conditions that recover the flavonoid triad efficiently, solvent polarity and temperature were screened. This study comprised two distinct extraction tracks, described separately below to avoid ambiguity between the analytical screening data and the extract used for biological testing.

#### 2.2.1. Track 1—Analytical Screening of Extraction Conditions

The optimization phase involved a comparative analysis of four solvent systems: 100% Water (DW), 30% Methanol, 30% Ethanol, and 100% Butanol. Each system was evaluated across a temperature gradient of 0, 10, 20, 30, and 50 °C, utilizing a solvent-to-solid ratio of 20:1 (*v*/*w*; 1 g of pulverized powder in 20 mL of solvent). The four solvents were selected to span a polarity range from water, through 30% hydroalcoholic mixtures, to a less polar solvent (100% butanol); alcohol concentrations other than 30% were not tested. All extractions were performed in three replicates. This track was designed to identify the condition giving the highest recovery of rutin, quercetin, and kaempferol among those tested. 30% ethanol at 50 °C gave the highest recovery.

#### 2.2.2. Track 2—Preparation of the Extract Used for Biological Testing

Room temperature (RT, ~20–25 °C) extraction was used to prepare the Mung Bean Sprout Extract (MSE) applied in all cell-based and animal experiments, independently of the Track 1 results. We hypothesized that RT extraction might better preserve thermolabile compounds; however, this was not experimentally compared with the 50 °C extract. The Sanpo cultivar (10-day germination) was extracted with 30% ethanol (20:1 *v*/*w*) under continuous stirring for 1 h at RT. The slurry was centrifuged at 7000 rpm for 10 min and filtered through a 0.22 μm PVDF membrane. The filtrate was concentrated under reduced pressure using a rotary evaporator and subsequently lyophilized to yield a stable powder, and stored at −20 °C.

Rationale for the RT/50 °C discrepancy: The 50 °C condition in Track 1 gave the highest rutin recovery but was not applied to the extract used for bioactivity testing; the biological data in this manuscript therefore pertain only to the RT extract. The 50 °C condition is a candidate for future scale-up, and the bioactivity of the 50 °C extract remains to be tested.

### 2.3. Phytochemical Profiling and Antioxidant Capacity Screening

For the cultivar screening, freeze-dried powder of Day 10 sprouts of each cultivar was extracted at a ratio of 1 g/10 mL with 80% methanol for total phenolic content (TPC) and total flavonoid content (TFC), and with 70% ethanol for DPPH and ABTS assays, at room temperature.

TPC was determined using the Folin–Ciocalteu colorimetric method (reagent: Sigma-Aldrich, St. Louis, MO, USA) [20]. The methanolic extract was diluted 30-fold before the assay. Absorbance was measured at 750 nm using a Varioskan Flash (Thermo Fisher Scientific, Waltham, MA, USA) and quantified against a gallic acid (≥98%, Sigma-Aldrich) calibration curve (y = 0.005x + 0.0578, R^2^ = 0.999), where y represents the absorbance and x denotes the concentration of gallic acid (μg/mL). All measurements were performed in three independent batches. Results were expressed as milligrams of gallic acid equivalents per gram of dry weight (mg GAE/g DW = (µg/mL ÷ 1000) × 10 × 30).

TFC was assessed via the aluminum chloride colorimetric assay using a catechin standard (≥98%, Sigma-Aldrich; calibration curve: y = 0.0021x + 0.0429, R^2^ = 0.998) and expressed as mg CE/g DW [21]. The methanolic extract was diluted 10-fold before the assay.

Antioxidant capacity was screened by DPPH (2,2-diphenyl-1-picrylhydrazyl, Sigma-Aldrich) and ABTS (2,2′-azino-bis(3-ethylbenzothiazoline-6-sulfonic acid), Sigma-Aldrich) radical scavenging assays using the 70% ethanol extracts. Extracts (1 g/10 mL, equivalent to 100 mg sprout powder DW/mL) were diluted 30-fold (DPPH) or 20-fold (ABTS), giving approximate final concentrations of 1.7 and 0.17 mg DW/mL in the reaction mixtures, respectively. For the DPPH assay, extracts were diluted 30-fold, and absorbance was read at 517 nm [22]. For the ABTS assay, extracts were diluted 20-fold to ensure that absorbance fell within the linear range of the standard curve, and absorbance was read at 732 nm [23]. Results are expressed as % radical scavenging and as equivalents of the standard used for the calibration curve—Trolox. Each extract was analyzed at a single dilution, and IC50 values were not determined. No reference antioxidant was included.

Sanpo was selected based on TPC and ABTS (see Section 3.1). TPC and TFC were measured only at Day 10 for cultivar screening and were not monitored across germination; flavonoid-specific tracking (Section 3.2) was used to select the harvest time. TPC and TFC were measured only at Day 10 and were not tracked across germination.

#### UHPLC-DAD Quantification and LC-MS Identification

Flavonoids were quantified by UHPLC-DAD (Thermo Ultimate 3000, (Thermo Fisher Scientific, Waltham, MA, USA); Acclaim RSLC PA2 PolarAdvantage II column, 2.2 µm, 2.1 × 150 mm; column 35 °C; autosampler 10 °C; injection 1 µL; flow 0.3 mL/min; detection at 350 nm). Mobile phase A was 0.1% formic acid in water, and B was 0.1% formic acid in acetonitrile; the gradient was 5% B (0–2 min), 25% B (20 min), 50% B (25 min), 90% B (30–33 min), and 5% B (37–40 min). Powder (15 mg) was extracted with 300 µL of solvent by shaking for 60 min at room temperature, centrifuged at 14,000 rpm for 10 min, and filtered through a 0.22 µm filter. Rutin (≥97%, PhytoLab GmbH & Co. KG, Vestenbergsgreuth, Germany), quercetin (≥99%, Sigma-Aldrich), and kaempferol (≥99%, PhytoLab GmbH & Co. KG) were quantified from peak areas using external calibration curves (five points; r^2^ ≥ 0.998) of authentic standards. Peak identity was confirmed by LC-ESI-MS/MS (CORTECS UPLC T3, Waters(Milford, MA, USA);1.6 µm, 2.1 × 150 mm; positive mode, *m*/*z* 100–1500) against the same standards. ND indicates no detectable peak at the retention time of the standard. LOD and LOQ were not determined; the method was used for relative comparison among cultivars, harvest days, and extraction conditions.

### 2.4. In Vitro Anti-Inflammatory and Cytotoxicity Assays

To evaluate the anti-inflammatory activity of MSE, it was tested using RAW 264.7 macrophages (American Type Culture Collection, ATCC, Manassas, VA, USA), HaCaT keratinocytes (Korean Cell Line Bank, Seoul, Republic of Korea), and YD-38 gingival cells (Korean Cell Line Bank). Cells were cultured in Dulbecco’s Modified Eagle Medium (DMEM; Gibco, Thermo Fisher Scientific, Waltham, MA, USA) or Roswell Park Memorial Institute (RPMI) 1640 medium (Gibco) supplemented with 10% fetal bovine serum (FBS; Gibco) and maintained at 37 °C in a humidified 5% CO_2_ atmosphere.

Cell viability was assessed using the EZ-Cytox assay kit (DoGenBio, Seoul, Republic of Korea) according to the manufacturer’s instructions. Initial cytotoxicity screening was performed up to 2000 μg/mL to establish a Wide Safety Margin (WSM); subsequently, sub-toxic concentrations (≤100 μg/mL) were selected for inflammatory modeling.

To induce inflammation, the following stimulation parameters were applied: RAW264.7-Lipopolysaccharide (LPS, from *Escherichia coli* O111:B4, Sigma-Aldrich) at 1 μg/mL, HaCaT- Combined TNF-α and IFN-γ (PeproTech, Cranbury, NJ, USA) at 30 ng/mL, YD-38- Combined TNF-α and IFN-γ at 20 ng/mL.

The release of pro-inflammatory cytokines (IL-6 and IL-8) was quantified via ELISA (R&D Systems, Minneapolis, MN, USA) using a Tecan Infinite 200Pro reader (Tecan Group Ltd., Männedorf, Switzerland). All treatments were performed in three biological replicates. To account for varying baselines, the anti-inflammatory efficacy was calculated using the following baseline-subtracted normalization formula:

Inhibition Rate (%) = [1 − (Csample − Ccontrol)/(Cstimulation − Ccontrol)] × 100

The anti-inflammatory responses observed justified the progression to an in vivo periodontitis model.

### 2.5. Animals, Periodontitis Induction, and Histological Evaluation

The physiological relevance of the MSE anti-inflammatory effect was evaluated using a *Porphyromonas gingivalis* LPS-induced periodontitis model [24]. The experimental unit in this study was a single animal. All procedures were approved by the Institutional Animal Care and Use Committee of Chonbuk National University Hospital (Institutional Effectiveness Evaluation Center; JBNUH-IACUC-2024-20, approved on 8 August 2024).

Animals and housing: Male Sprague-Dawley rats were obtained at 6 weeks of age from Koatech (Pyeongtaek, Republic of Korea; total n = 35 received), acclimated for 1 week, and used at 7 weeks of age (n = 7 per group for the three arms reported here; the remaining 14 animals were assigned to other test groups of the same study and are not reported here). Animals were experimentally naïve prior to the study, with no prior procedures performed. Animals were housed in an SPF facility (Room No. 1) under controlled conditions: temperature 20–26 °C, relative humidity 40–70%, ventilation 12 air changes/h, a 12 h light/12 h dark cycle (lights on 08:00–20:00), and illuminance of 150–300 lux, in polycarbonate cages (310W × 500D × 200H mm; ≤5 animals/cage). Animals had free access to standard rodent chow (Orient Bio Inc., Seongnam, Republic of Korea) and water; feed quality was verified via periodic certificates of analysis from the supplier. Following acclimation, animals judged healthy were ranked by body weight and randomly allocated to groups such that group mean body weights were balanced. No animals were excluded from the study or from the analysis; all animals that passed the health check during acclimation were included, and no a priori exclusion criteria were defined.

Periodontitis induction: Under anesthesia (ketamine 75 mg/kg + xylazine 10 mg/kg, i.p.), *P. gingivalis* LPS (Sigma-Aldrich; 1 mg/mL in sterile saline, 1 μL) was injected into the buccal/lingual gingiva at the neck of the maxillary first molar using a Hamilton microsyringe (Hamilton Company, Reno, NV, USA), and the injection was repeated identically 3 days later. Normal control animals (G1) received an equal volume of sterile saline via the same procedure.

Test article administration: MSE was weighed on a crude dry-weight basis (without correction for active-compound content) and suspended in 1% high-viscosity carboxymethylcellulose (CMC; Sigma-Aldrich) in distilled water, freshly prepared by vortex mixing on the day of dosing. The dose (50 mg/kg/day) was pre-specified per the study sponsor’s protocol requirements. Treatment was administered once daily (~10:00 AM) via oral gavage (disposable feeding tube) for 10 consecutive days; G1 and G2 received vehicle only. General clinical observations were recorded twice daily (morning/afternoon); body weight was measured at intake and at group allocation. No humane endpoints were pre-defined for this study, and no unexpected adverse events or mortality were observed during the experimental period.

Histopathology: Following euthanasia, maxillae containing the first molar were excised, fixed in 10% neutral buffered formalin for 24 h, decalcified in Decalcifying Solution (Sigma-Aldrich, St. Louis, MO, USA) for 3 days, and paraffin-embedded. Histological evaluation was performed on the resulting decalcified, paraffin-embedded sections stained with hematoxylin and eosin (H&E). Periodontal inflammation and alveolar bone loss were assessed under a light microscope (Olympus, Tokyo, Japan). Quantitative histomorphometry—cementoenamel junction (CEJ) to alveolar bone crest (ABC) distance—was performed using Image-Pro software (Media Cybernetics, Rockville, MD, USA), under blinded conditions. Semi-quantitative periodontal inflammation scoring (PD score, 0–4 scale) was performed according to the criteria of Aguirre et al. [25]. No study protocol was prepared or registered in a public registry prior to the commencement of this study.

### 2.6. Statistical Analysis

Data are expressed as mean ± SD, or as median and interquartile range (IQR) for ordinal data (PD score). Statistical analyses were performed using GraphPad Prism (v11.1.0, GraphPad Software, Boston, MA, USA). Normality and homogeneity of variance were assessed using the Shapiro–Wilk and Levene tests, respectively. Cultivar screening data (TPC, TFC, DPPH, and ABTS) were compared by one-way ANOVA followed by Tukey’s post hoc test. Flavonoid contents among germination days were compared within each sample by one-way ANOVA followed by Tukey’s test. In vitro data were compared by one-way ANOVA with Tukey’s post hoc test. For the in vivo study, CEJ-ABC distance was compared among the three groups by one-way ANOVA with Tukey’s post hoc test, and the PD score (an ordinal scale) was compared using the Kruskal–Wallis test with Dunn’s post hoc test (Bonferroni-adjusted); a Kruskal–Wallis test was also applied to CEJ-ABC distance as a sensitivity analysis. Statistical significance was defined as *p* < 0.05. All in vitro experiments were performed in at least three independent replicates.

## 3. Results

### 3.1. Comparative Screening of Bioactive Potentials in Domestic Mung Bean Cultivars

**Figure 1:**
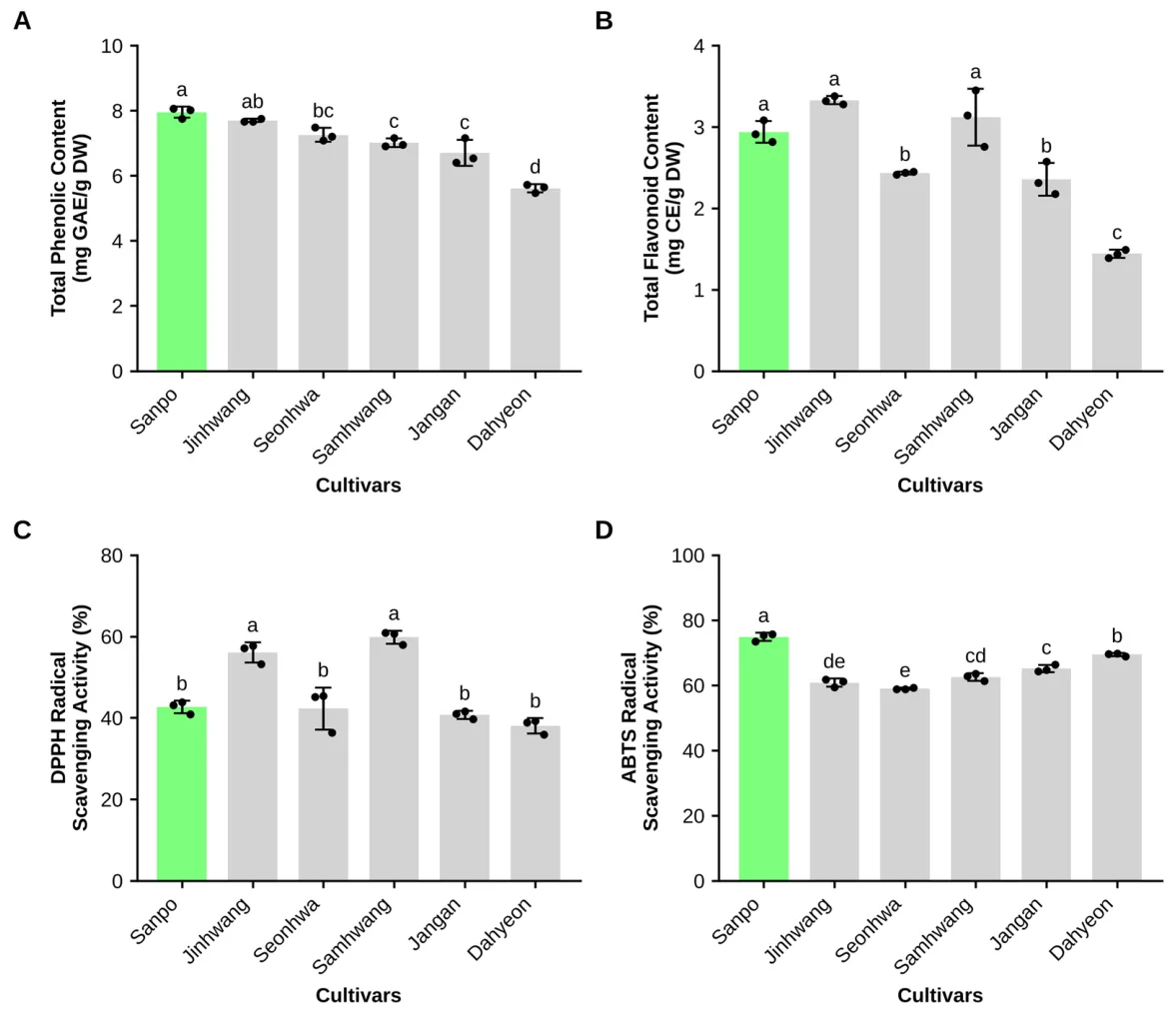
Phenolic content and radical scavenging activities of six mung bean cultivars. (**A**) Total phenolic content (TPC, mg GAE/g DW). (**B**) Total flavonoid content (TFC, mg CE/g DW). (**C**) DPPH radical scavenging activity (%). (**D**) ABTS radical scavenging activity (%). Bars show mean ± SD with individual values (n = 3 independent extractions). Different letters indicate significant differences among cultivars (one-way ANOVA with Tukey’s test, *p* < 0.05). The lead cultivar ‘Sanpo’ is highlighted in green.

To select a cultivar for subsequent experiments, six domestic mung bean cultivars were compared for TPC, TFC, and DPPH and ABTS radical scavenging activities (Table 1 and Figure 1). All four parameters differed significantly among cultivars (one-way ANOVA, *p* < 0.001). Sanpo had the highest TPC, which did not differ significantly from that of Jinhwang, and the highest ABTS activity, which was significantly higher than that of all other cultivars. In contrast, the TFC of Sanpo was comparable to that of Jinhwang and Samhwang, and its DPPH activity was significantly lower than that of Jinhwang and Samhwang. Because DPPH and ABTS assays probe different mechanisms and gave different rankings, Sanpo was selected on the basis of its TPC and ABTS values.

### 3.2. Harvest-Time Profiling of Flavonoid Content

**Figure 2:**
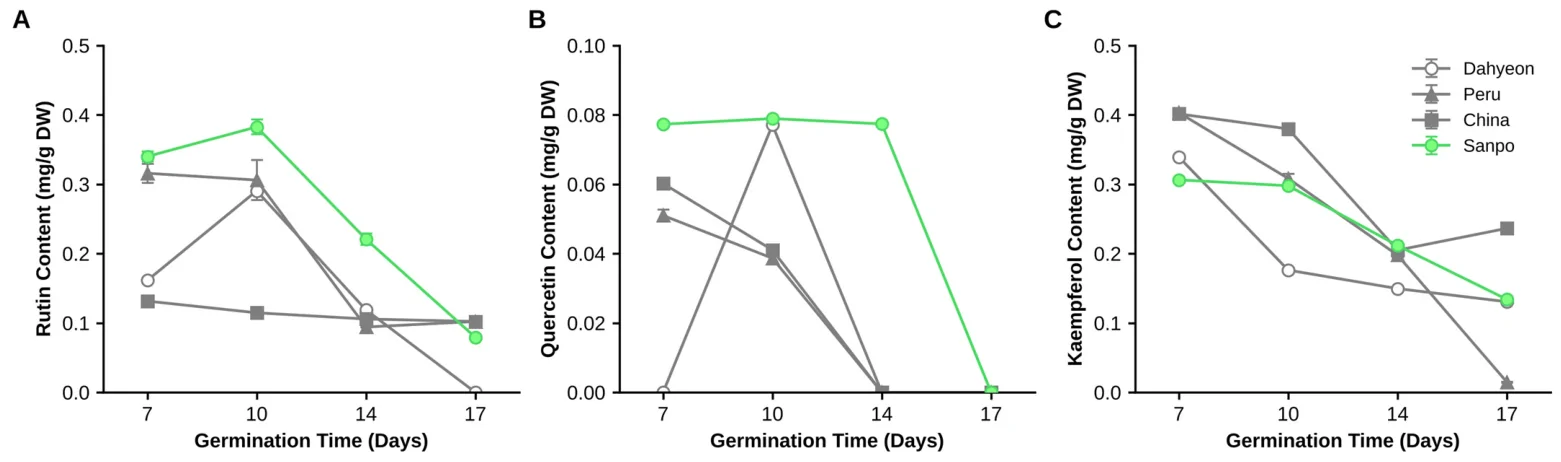
Developmental kinetics of target flavonoid triad during germination. Rutin (**A**), quercetin (**B**), and kaempferol (**C**) contents (mg/g DW) of Sanpo, Dahyeon, and the imported Peru and China sprouts at germination Days 7, 10, 14, and 17. Data are means ± SD (n = 3 independent extractions). Day 10 was selected as the harvest time for Sanpo.

The metabolic trajectory of target flavonoids—rutin, quercetin, and kaempferol—was monitored over a 17-day germination period (Days 7, 10, 14, and 17) to identify a suitable harvest time for the lead cultivar, Sanpo, alongside Dahyeon and the imported Peru and China accessions (Figure 2 and Table S1).

In Sanpo, rutin was significantly higher at Day 10 than at Days 7, 14, and 17 (one-way ANOVA with Tukey’s test, *p* < 0.001), whereas kaempferol was highest at Day 7 (*p* = 0.007 vs. Day 10). Quercetin differed by less than 0.002 mg/g DW among Days 7–14. Day 10 Sanpo sprouts had the highest rutin content (0.3825 mg/g DW) and the highest quercetin content (0.0789 mg/g DW), with kaempferol at 0.2977 mg/g DW (Figure 2 and Table S1). Kaempferol was highest at Day 7 (0.3062 mg/g DW), and quercetin in Sanpo was similar at Days 7 and 14 (0.0773 and 0.0774 mg/g DW) before becoming undetectable at Day 17.

In Dahyeon and the imported Peru and China samples, quercetin was undetectable by Day 14. Day 10 was therefore selected as the harvest time at which rutin was highest, and quercetin was still present; differences from adjacent time points were small (mean of three independent extractions; Table S1).

### 3.3. Extraction Screening: Analytical Conditions Versus the Extract Used for Biological Testing

Consistent with the two-track extraction design (see Methods 2.2), analytical screening of solvent systems and thermal kinetics was systematically performed to identify the condition giving the highest recovery of the target flavonoids, distinct from the RT-extracted MSE used for biological testing.

**Figure 3:**
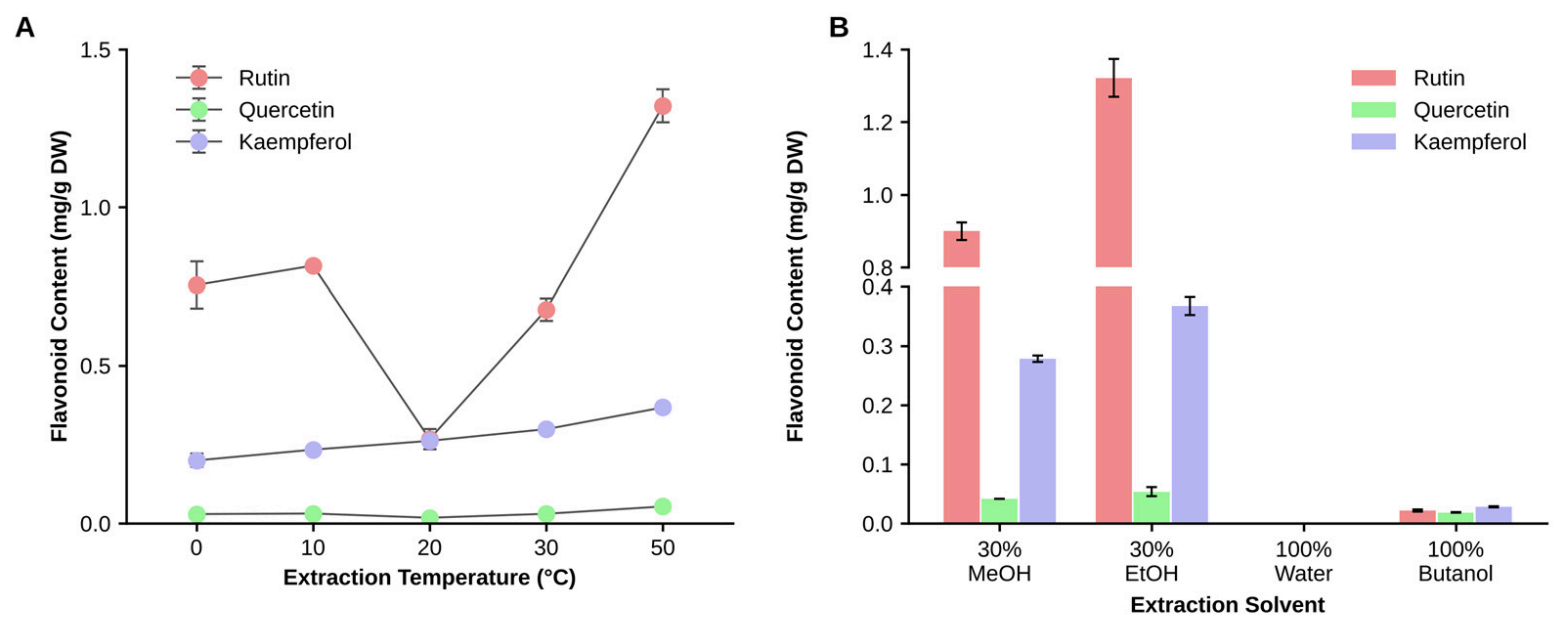
Effect of extraction temperature and solvent on flavonoid recovery in Sanpo. (**A**) Rutin, quercetin, and kaempferol contents in 30% ethanol at 0–50 °C. (**B**) Flavonoid recovery at 50 °C with different solvent systems (30% MeOH, 30% EtOH, 100% Water, and 100% Butanol). Data are mean ± SD (n = 3 independent extractions; mg/g DW).

In Sanpo, rutin recovery in 30% ethanol was highest at 50 °C (1.3208 mg/g DW), but it did not increase monotonically across the temperature series (0.7540, 0.8150, 0.2670, 0.6766, and 1.3208 mg/g DW at 0, 10, 20, 30, and 50 °C; Table 2, Figure 3A and Table S2). Quercetin and kaempferol at 50 °C were 0.0535 and 0.3672 mg/g DW, respectively. At 20 °C, the condition closest to the RT extraction used for MSE, rutin, quercetin, and kaempferol were 0.2670, 0.0178, and 0.2613 mg/g DW. Recovery was also highest at 50 °C in the other five cultivars (Table S2).

Recovery at 20 °C was lower than at the adjacent temperatures in five of the six cultivars (Table S2), which may reflect run-to-run variability in extraction or analysis; the conclusion that 50 °C gave the highest recovery was unaffected. At 50 °C, 30% ethanol gave higher recovery (rutin: 1.3208 mg/g DW, quercetin: 0.0535 mg/g DW, kaempferol: 0.3672 mg/g DW) than 30% methanol (rutin: 0.8995 mg/g DW, quercetin: 0.0412 mg/g DW, kaempferol: 0.2783 mg/g DW) (Figure 3B). In contrast, extraction with 100% water failed to recover detectable levels of any of the three target flavonoids, while 100% butanol yielded negligible recoveries (rutin: 0.0215 mg/g DW, quercetin: 0.0184 mg/g DW, kaempferol: 0.0279 mg/g DW) (Figure 3B). 30% ethanol at 50 °C gave the highest recovery under the conditions tested; whether this condition retains the biological activity of the RT extract was not tested.

### 3.4. In Vitro Cytocompatibility and Anti-Inflammatory Activity in Three Cell Models

**Figure 4:**
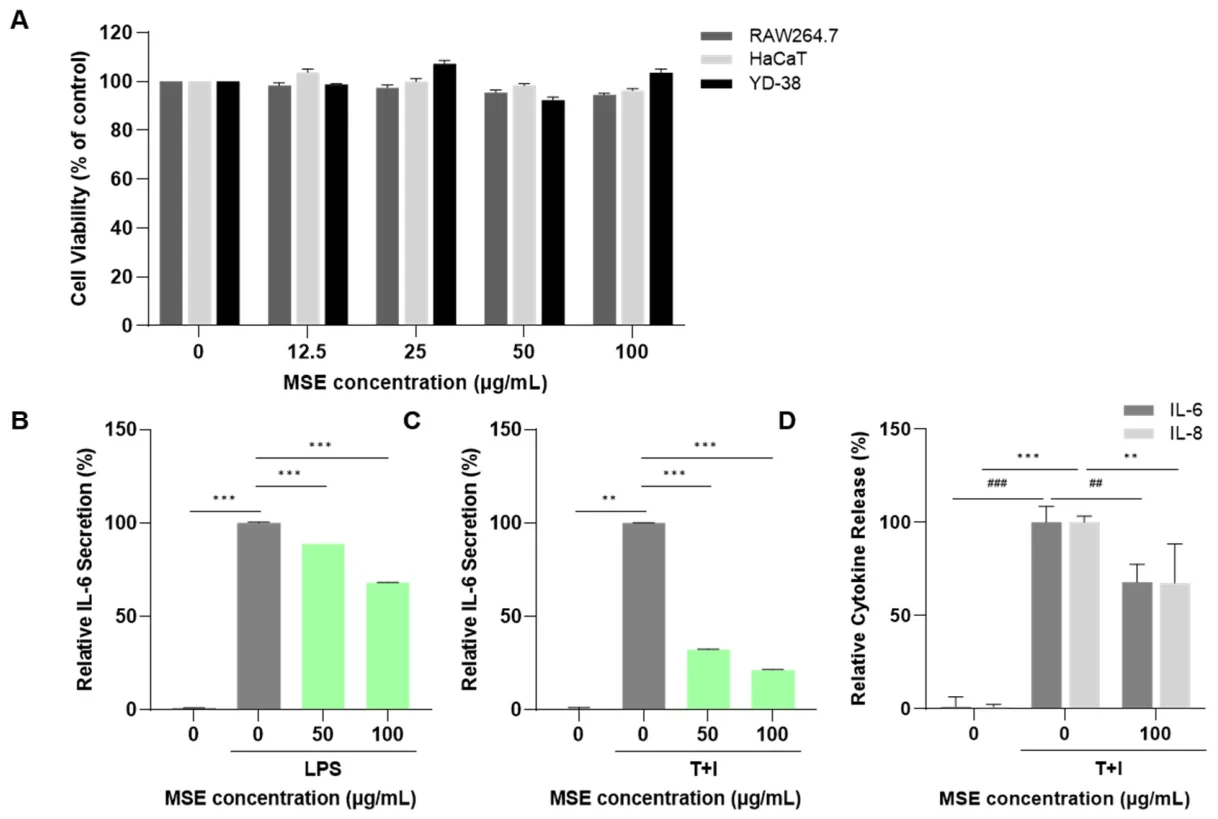
In vitro multi-barrier cytocompatibility and anti-inflammatory activity. (**A**) Cell viability (% of control) in RAW264.7, HaCaT, and YD-38 cells treated with MSE (0–100 μg/mL). (**B**) Relative IL-6 secretion in LPS-stimulated RAW264.7 macrophages. (**C**) Relative IL-6 secretion in TNF-α/IFN-γ-stimulated HaCaT keratinocytes. (**D**) Relative cytokine release (IL-6 and IL-8) in TNF-α/IFN-γ-stimulated YD-38 gingival cells. Data are presented as baseline-subtracted inhibition levels. Asterisks indicate significant differences between the groups connected by lines (** *p* < 0.01, *** *p* < 0.001); in panel D, asterisks indicate IL-8 and hash symbols indicate IL-6 (^##^ *p* < 0.01, ^###^ *p* < 0.001). Light green bars in panels B and C indicate MSE-treated groups. T+I, TNF-α/IFN-γ.

To evaluate the cytocompatibility and anti-inflammatory activity of Sanpo MSE in macrophage and epithelial cell models, initial cell viability screening was conducted on RAW264.7 murine macrophages, HaCaT human epidermal keratinocytes, and YD-38 human oral gingival epithelial cells (Figure 4A). Treatment with MSE at concentrations ranging from 0 to 100 μg/mL for 24 h resulted in no observable cytotoxicity, with cell viability consistently exceeding 90% across all three cell lines (RAW264.7: 94.6 ± 0.8%, HaCaT: 97.2 ± 0.6%, YD-38: 103.8 ± 1.7% at 100 μg/mL; Figure 4A). This confirmed a wide safety margin for downstream inflammatory modeling.

In LPS-stimulated RAW264.7 macrophages (1 μg/mL), baseline IL-6 secretion increased markedly from 1601.3 ± 3.2 pg/mL (CON) to 4303.9 ± 12.5 pg/mL (LPS control). Treatment with MSE at 100 μg/mL significantly attenuated IL-6 release to 3443.6 ± 18.2 pg/mL, representing a 31.95% suppression of the pure stimulated inflammatory response (*p* < 0.001 vs. LPS; Figure 4B).

In HaCaT keratinocytes co-stimulated with recombinant TNF-α and IFN-γ (T+I, 30 ng/mL), baseline IL-6 rose from 109.4 ± 0.1 pg/mL (CON) to 142.5 ± 0.1 pg/mL (T+I control). MSE demonstrated a dose-dependent reduction, reducing IL-6 concentrations to 120.2 ± 0.1 pg/mL at 50 μg/mL and 116.5 ± 0.1 pg/mL at 100 μg/mL. Under the baseline-subtracted normalization model, MSE (100 μg/mL) achieved a 77.68% inhibition of pure T+I-induced IL-6 elevation (*p* < 0.001; Figure 4C).

Furthermore, in YD-38 oral gingival epithelial cells co-stimulated with T+I (20 ng/mL), MSE (100 μg/mL) displayed dual cytokine-suppressive activity, simultaneously reducing IL-6 from 1031.0 ± 88.7 pg/mL to 807.0 ± 99.1 pg/mL (32.05% inhibition, *p* < 0.01) and IL-8 from 1133.0 ± 37.7 pg/mL to 887.0 ± 237.3 pg/mL (32.54% inhibition, *p* < 0.01; Figure 4D). These results indicate that Sanpo MSE reduced IL-6 and IL-8 release in macrophage, keratinocyte, and gingival epithelial cell models under the conditions tested.

### 3.5. In Vivo Effects of MSE in a Periodontitis Rat Model

**Figure 5:**
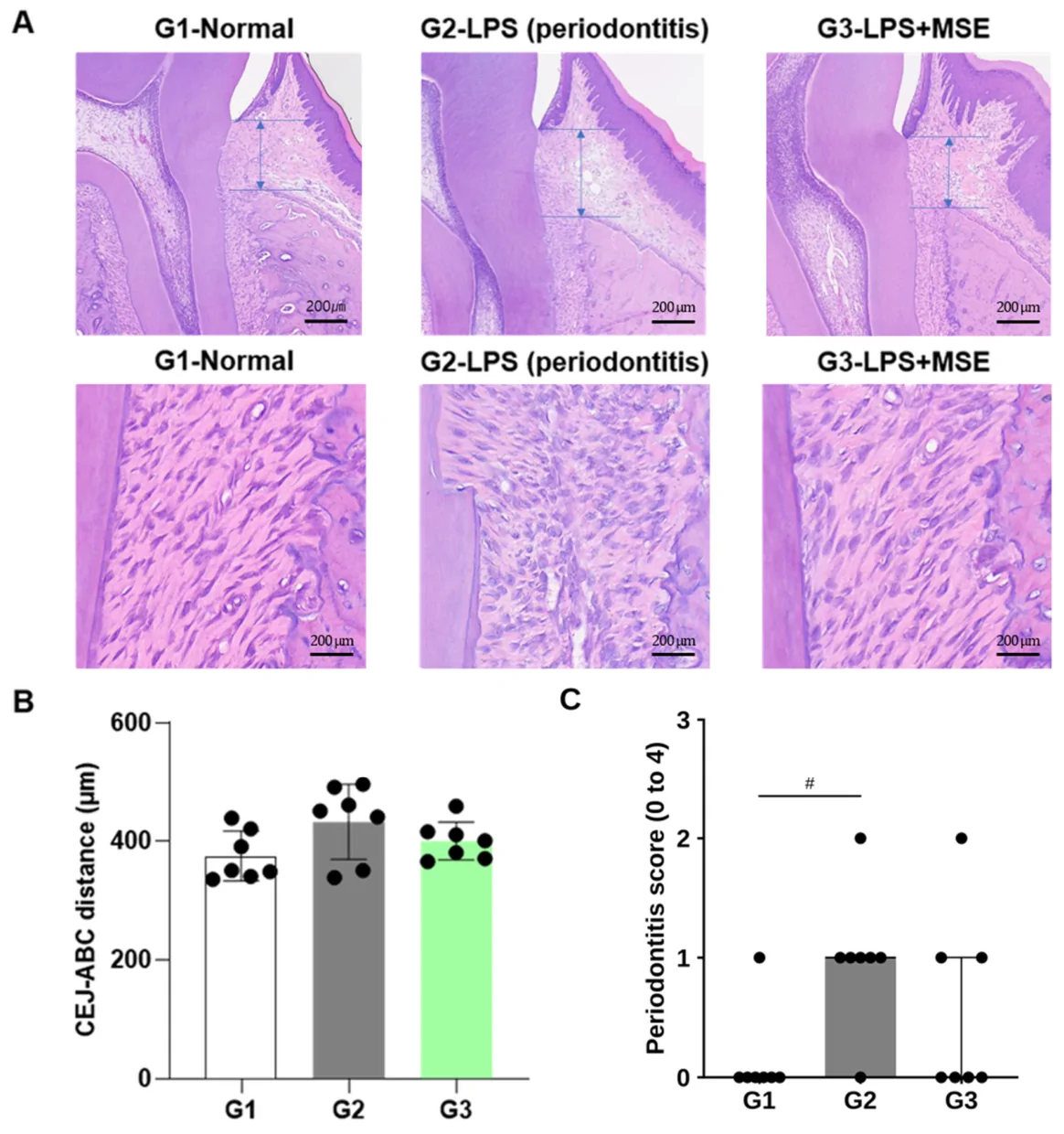
In vivo effects of MSE on alveolar bone loss and periodontal histology in a *P. gingivalis* LPS-induced periodontitis rat model. (**A**) Representative H&E-stained histological sections of maxillary molars showing cementoenamel junction-alveolar bone crest (CEJ-ABC) distance (top row; double-headed arrows indicate the CEJ-ABC distance; scale bar = 200 µm) and periodontal ligament (PDL) fiber alignment (bottom row) across groups: G1 (Normal), G2 (LPS-induced periodontitis), and G3 (LPS + MSE 50 mg/kg/day). (**B**) Quantitative histomorphometric measurement of CEJ-ABC distance (µm). (**C**) Semi-quantitative periodontal inflammation scores (0–4 scale). Data are mean ± SD for panel B and median (IQR) for panel (**C**) (n = 7 per group; dots in panels B and C, individual animals). In panel (**C**), bars indicate medians and whiskers indicate IQR (no whisker is visible where the IQR is zero-width). CEJ-ABC distance was analyzed by one-way ANOVA with Tukey’s test (no significant differences); PD score by Kruskal–Wallis test with Dunn’s test. # *p* < 0.05, G2 vs. G1 (panel (**C**) only).

The effects of Sanpo MSE on alveolar bone loss and periodontal inflammation were evaluated in a *Porphyromonas gingivalis* LPS-induced periodontitis Sprague-Dawley rat model (n = 7 per group), evaluating Normal vehicle control (G1), LPS-induced periodontitis model (G2), and LPS + MSE oral treatment (50 mg/kg/day, G3) (Figure 5).

Quantification of the cementoenamel junction to alveolar bone crest (CEJ-ABC) distance showed a tendency toward greater alveolar bone loss in the LPS group (G2: 427.6 ± 60.3 µm) than in the normal control group (G1: 375.0 ± 46.2 µm; Tukey *p* = 0.127), although the difference was not statistically significant (Figure 5A,B). The MSE-treated group (G3) showed a numerically lower CEJ-ABC distance (401.3 ± 32.8 µm) than G2, which was also not statistically significant (*p* = 0.567) (Figure 5A,B).

Periodontal inflammation, assessed by the PD score (0–4 scale), was significantly higher in the LPS group (G2: median 1 [IQR 1–1]) than in the normal group (G1: 0 [0–0]; Dunn’s test, *p* = 0.035) (Figure 5C). The PD score in the MSE-treated group (G3: 0 [0–1]) was numerically lower than in G2 but did not differ significantly (*p* = 0.511).

Histopathological examination via hematoxylin and eosin (H&E) staining showed qualitative differences in periodontal ligament fiber alignment and leukocyte infiltration between groups (Figure 5A). Maxillary tissues from G2 exhibited marked intraepithelial inflammatory cell infiltration, increased cellularity in the lamina propria, and irregular disruption of periodontal ligament (PDL) cell alignment. In representative sections, MSE-treated rats (G3) appeared to show reduced leukocyte infiltration, a better-preserved alveolar crest architecture, and a more regular arrangement of collagenous PDL fibers (Figure 5A), although these observations were qualitative and not quantified.

## 4. Discussion

Sanpo showed the numerically highest TPC among the six cultivars (comparable to Jinhwang) and the highest ABTS radical-scavenging activity (74.85 ± 1.23%), but ranked third in the DPPH assay, and its TFC was comparable to Jinhwang and Samhwang. DPPH and ABTS differ in mechanism (predominantly single electron transfer in an organic medium versus hydrogen atom/electron transfer in aqueous-organic phases), and their rankings are known to diverge across plant matrices [18]. We therefore selected Sanpo on its combined TPC/ABTS profile, which may be more relevant to the aqueous conditions of the subsequent cell assays; this is a selection criterion, not a claim of superiority across all assays. The antioxidant results should be interpreted cautiously because each assay used a single dilution, IC50 values were not determined, and no reference antioxidant was included. The antioxidant activity is consistent with the presence of phenolic hydroxyl groups capable of hydrogen donation [26], but this was not tested directly.

The profiles of the three flavonoids over the 17-day germination period differed among compounds. In Sanpo, rutin was highest at Day 10, kaempferol was highest at Day 7 and declined thereafter, and quercetin was similar at Days 7–14 before becoming undetectable at Day 17; in Dahyeon and the imported accessions, quercetin was undetectable by Day 14. Quercetin may be generated transiently from rutin by glycosidase-mediated cleavage [17], but we did not measure enzyme activity. Day 10 was chosen as a time point at which rutin was highest, and quercetin remained detectable. Because these were measured in triplicate and the differences from neighbouring time points were small, the choice of Day 10 should be regarded as provisional. The flavonoid content of the lyophilized MSE itself was not measured; the 20 °C values in Table 2 are the closest available estimate. The extraction yield of the lyophilized MSE was also not recorded.

In Sanpo, rutin recovery in 30% ethanol was highest at 50 °C but did not increase monotonically across 0–50 °C; 20 °C, the condition closest to the RT extraction used for MSE, gave lower recovery (0.2670 mg/g DW) than 50 °C (1.3208 mg/g DW). Higher temperature may improve mass transfer and solubilization [19], but this was not examined here. The biological data were generated with the RT extract; we speculated that RT extraction may preserve thermolabile compounds, but this was not compared experimentally with the 50 °C extract, and the biological equivalence of the two extracts has not been tested. The 50 °C condition is therefore a candidate for future scale-up, and this comparison is a priority for future work.

Sanpo MSE reduced IL-6/IL-8 release in macrophage, keratinocyte, and gingival epithelial cell models, with cell viability exceeding 90% at concentrations up to 100 μg/mL [27]. This study did not include direct molecular measurements, such as Western blot or qPCR, of TLR4, NF-κB, or MAPK pathway components; the candidate mechanisms discussed here are therefore literature-based hypotheses consistent with our functional, cytokine-level observations, rather than directly confirmed findings. TLR4-mediated signaling may plausibly be modulated by MSE [28], given that LPS—a canonical TLR4 ligand—was the RAW264.7 stimulus; quercetin itself, one of the flavonoid triad’s constituents, has been directly shown to suppress NF-κB and MAPK (p38, ERK, JNK) phosphorylation in LPS-stimulated RAW264.7 macrophages [12], and a rutin-derived metabolite has likewise been shown to attenuate LPS-induced NF-κB activation in the same cell model [13]. We recommend that future studies incorporate direct protein- or transcript-level readouts to test these hypotheses for the present extract.

Oral MSE (50 mg/kg/day, 10 days) was associated with a numerically intermediate CEJ-ABC distance (401.3 ± 32.8 µm) relative to the LPS group (427.6 ± 60.3 µm) and the normal group (375.0 ± 46.2 µm), together with a lower median PD score (0 vs. 1 in G2) and qualitatively improved periodontal ligament fiber alignment on H&E staining [29]. The PD score was significantly higher in the LPS group than in the normal group (*p* = 0.035), whereas the corresponding CEJ-ABC difference did not reach significance (*p* = 0.127); the LPS vs. LPS+MSE comparison was not statistically significant for either endpoint [30]. We interpret these findings as hypothesis-generating, proof-of-concept evidence of a numerical trend rather than as confirmed therapeutic efficacy. This study used a single, sponsor-specified dose level rather than a dose derived from a formal dose-ranging study, and a modest per-group sample size (n = 7) consistent with exploratory rodent pilot designs; combined with the variance observed in the LPS group (SD = 60.3 µm), the study was likely underpowered to detect a statistically significant treatment effect even if a true biological effect exists. We present these in vivo findings as preliminary and recommend that future work include a dose-ranging design with an increased per-group sample size to confirm and more precisely define the therapeutic window. In addition, because the LPS-induced increase in CEJ-ABC distance was itself not statistically significant, this study has limited ability to evaluate a treatment effect on alveolar bone loss.

## 5. Conclusions

This study characterizes Sanpo mung bean sprout extract, covering cultivar screening, harvest-time profiling (Day 10), and extraction solvent/temperature screening, together with evaluation of the RT-extracted MSE in three in vitro cell models and a pilot periodontitis rat model. The RT-extracted MSE (30% ethanol) demonstrated cytocompatibility and significant anti-inflammatory activity (cytokine suppression) across immune, epidermal, and gingival cell models in vitro. In a single-dose (50 mg/kg/day), exploratory in vivo pilot study (n = 7/group), MSE was associated with a non-significant numerical trend toward reduced alveolar bone loss and periodontal inflammation relative to the LPS-only group; this trend did not reach statistical significance and should be regarded as hypothesis-generating rather than confirmatory.

We note the following as priorities for future validation prior to translational application: (i) a dose-ranging in vivo study with an increased per-group sample size to confirm and statistically establish the in vivo effect suggested by this pilot data; (ii) confirmation that the 50 °C extract retains the bioactivity profile established for the RT extract; and (iii) direct molecular pathway confirmation (e.g., Western blot/qPCR) of the proposed TLR4/NF-κB/MAPK axis. With these caveats, Sanpo MSE represents a promising candidate ingredient—supported by in vitro efficacy and a preliminary in vivo signal—for further development toward oral-care nutraceutical and functional pet-treat applications.

## Figures and Tables

**Table 1:** Phenolic and flavonoid contents and radical scavenging activities of six mung bean cultivars.

Cultivar	TPC (mg GAE/g DW)	TFC (mg CE/g DW)	DPPH (%)	ABTS (%)
Sanpo	7.95 ± 0.17 ^a^	2.94 ± 0.13 ^a^	42.62 ± 1.52 ^b^	74.85 ± 1.23 ^a^
Jinhwang	7.70 ± 0.05 ^ab^	3.33 ± 0.05 ^a^	56.01 ± 2.49 ^a^	60.79 ± 1.21 ^de^
Seonhwa	7.26 ± 0.21 ^bc^	2.43 ± 0.02 ^b^	42.28 ± 5.16 ^b^	58.97 ± 0.29 ^e^
Samhwang	7.01 ± 0.14 ^c^	3.12 ± 0.35 ^a^	59.82 ± 1.59 ^a^	62.53 ± 1.19 ^cd^
Jangan	6.70 ± 0.41 ^c^	2.35 ± 0.20 ^b^	40.75 ± 1.01 ^b^	65.12 ± 1.12 ^c^
Dahyeon	5.61 ± 0.13 ^d^	1.44 ± 0.05 ^c^	38.01 ± 1.86 ^b^	69.38 ± 0.47 ^b^

Values are mean ± SD (n = 3 independent extractions). Different letters within a column indicate significant differences (one-way ANOVA followed by Tukey’s HSD test, *p* < 0.05). TPC, total phenolic content; TFC, total flavonoid content; GAE, gallic acid equivalents; CE, catechin equivalents; DW, dry weight.

**Table 2:** Rutin, quercetin, and kaempferol contents (mg/g DW) of Sanpo sprouts extracted with 30% ethanol at different temperatures.

Compound	0 °C	10 °C	20 °C	30 °C	50 °C
Rutin	0.754 ± 0.075 ^bc^	0.815 ± 0.009 ^b^	0.267 ± 0.032 ^d^	0.677 ± 0.035 ^c^	1.321 ± 0.052 ^a^
Quercetin	0.029 ± 0.002 ^b^	0.031 ± 0.000 ^b^	0.018 ± 0.001 ^c^	0.030 ± 0.002 ^b^	0.054 ± 0.008 ^a^
Kaempferol	0.199 ± 0.021 ^d^	0.234 ± 0.007 ^c^	0.261 ± 0.006 ^c^	0.299 ± 0.002 ^b^	0.367 ± 0.015 ^a^

Values are mean ± SD (n = 3 independent extractions; mg/g DW). Different letters within a row indicate significant differences among temperatures (one-way ANOVA followed by Tukey’s HSD test, *p* < 0.05).

## Data Availability

The data presented in this study are available on request from the corresponding author.

## Supplementary Materials

The following supporting information can be downloaded at: https://www.mdpi.com/article/10.3390/foods15213910/s1, Table S1: Developmental changes in flavonoid contents of mung bean sprout cultivars across germination days (mg/g DW, mean ± SD); Table S2: Rutin contents of six mung bean cultivars extracted with 30% ethanol or 30% methanol at different temperatures (mg/g DW, mean ± SD, n = 3).
