# Supplementary material for: Phytochemical Characterization of Germinated Mung Bean Sprout (Vigna radiata L., cv. Sanpo) and Anti-Inflammatory Activity In Vitro and Preliminary Effects in a Rat Periodontitis Model
Source: Foods. 2026 Oct 31;15(21):3910. doi: 10.3390/foods15213910 (PMC13763475; doi:10.3390/foods15213910)
Supplement: Supplementary file 1 [file foods-15-03910-s001.zip › foods-4596942-supplementary.pdf]

[Supplementary Materials]

Phytochemical Characterization of Germinated Mung Bean Sprout (*Vigna radiata* L., cv. Sanpo) and Anti-Inflammatory Activity In Vitro and Preliminary Effects in a Rat Periodontitis Model

Ji Yeong Yang [1], Sang Hoon Lee [2], Yeong Jae An [2], Se Won Cho [2] and Seung Yeob Song [2,*]

[1] Department of Clinical Laboratory Science, Dongnam Health University, Suwon 16328, Republic of Korea; jyyang@dongnam.ac.kr

[2] Food Tech Resources Research Division, Department of Food Sciences, National Institute of Crop and Food Science, Wanju 55365, Republic of Korea; spprigan@korea.kr (S.H.L.); ayj55381@naver.com (Y.J.A.); fsw1208@naver.com (S.W.C.)

* Correspondence: s2y337@korea.kr; Tel.: +82-(0)63-238-3698; Fax: +82-(0)63-238-3842

Table S1. Developmental Changes in Flavonoid Contents of Mung Bean Sprout Cultivars Across Germination Days (mg/g DW, mean ± SD).

Cultivar	Compound	DAY 7	DAY 10	DAY 14	DAY 17
Sanpo	Rutin	0.340 ± 0.007	0.382 ± 0.011	0.221 ± 0.008	0.079 ± 0.000
	Quercetin	0.077 ± 0.001	0.079 ± 0.000	0.077 ± 0.000	ND
	Kaempferol	0.306 ± 0.002	0.298 ± 0.003	0.211 ± 0.002	0.134 ± 0.001
Dahyeon	Rutin	0.161 ± 0.004	0.290 ± 0.005	0.119 ± 0.001	ND
	Quercetin	ND	0.077 ± 0.001	ND	ND
	Kaempferol	0.339 ± 0.004	0.176 ± 0.000	0.149 ± 0.002	0.130 ± 0.001
Peru (imported)	Rutin	0.316 ± 0.014	0.306 ± 0.029	0.094 ± 0.001	0.102 ± 0.006
	Quercetin	0.051 ± 0.002	0.039 ± 0.002	ND	ND
	Kaempferol	0.403 ± 0.001	0.308 ± 0.007	0.198 ± 0.003	0.015 ± 0.001
China (imported)	Rutin	0.131 ± 0.006	0.115 ± 0.001	0.106 ± 0.003	0.102 ± 0.002
	Quercetin	0.060 ± 0.000	0.041 ± 0.000	ND	ND
	Kaempferol	0.401 ± 0.003	0.380 ± 0.003	0.205 ± 0.002	0.236 ± 0.004

Values are mean ± SD (n = 3 independent extractions). ND, not detected. Peru and China are imported accessions. Peru Day 17 quercetin was detected in one of three replicates and is reported as ND.

Table S2. Rutin Contents of Six Mung Bean Cultivars Extracted with 30% Ethanol or 30% Methanol at Different Temperatures (mg/g DW, mean ± SD, n = 3).

Cultivar	Solvent	0°C	10°C	20°C	30°C	50°C
Sanpo	30% EtOH	0.754 ± 0.075	0.815 ± 0.009	0.267 ± 0.032	0.677 ± 0.035	1.321 ± 0.052
	30% MeOH	0.484 ± 0.018	0.429 ± 0.007	0.031 ± 0.008	0.303 ± 0.057	0.900 ± 0.024
Dahyeon	30% EtOH	0.316 ± 0.008	0.422 ± 0.015	0.494 ± 0.023	0.600 ± 0.056	0.653 ± 0.027
	30% MeOH	0.248 ± 0.007	0.322 ± 0.010	0.398 ± 0.028	0.447 ± 0.025	0.485 ± 0.016
Jinhwang	30% EtOH	0.441 ± 0.051	0.468 ± 0.031	0.320 ± 0.005	0.511 ± 0.017	0.896 ± 0.019
	30% MeOH	0.273 ± 0.006	0.268 ± 0.043	0.205 ± 0.033	0.348 ± 0.009	0.600 ± 0.006
Samhwang	30% EtOH	0.655 ± 0.010	0.603 ± 0.037	0.397 ± 0.035	0.734 ± 0.016	1.132 ± 0.035
	30% MeOH	0.358 ± 0.010	0.370 ± 0.025	0.280 ± 0.014	0.423 ± 0.014	0.781 ± 0.010
Seonhwa	30% EtOH	0.369 ± 0.003	0.348 ± 0.007	0.242 ± 0.016	0.363 ± 0.019	0.605 ± 0.003
	30% MeOH	0.237 ± 0.009	0.206 ± 0.020	0.153 ± 0.005	0.238 ± 0.016	0.454 ± 0.018
Jangan	30% EtOH	0.385 ± 0.042	0.353 ± 0.026	0.182 ± 0.009	0.362 ± 0.005	0.674 ± 0.026
	30% MeOH	0.283 ± 0.010	0.218 ± 0.021	0.035 ± 0.010	0.229 ± 0.006	0.517 ± 0.020

100% water: ND in all cultivars and at all temperatures. 100% butanol: ND at 0–20 °C and ≤ 0.021 mg/g DW at 30–50 °C in all cultivars (n = 3). ND, not detected.
